# Capecitabine-induced cardiotoxicity: case report and review of the literature

**DOI:** 10.3747/co.v17i1.437

**Published:** 2010-02

**Authors:** C. Ang, M. Kornbluth, M.P. Thirlwell, R.D. Rajan

**Affiliations:** * Department of Oncology, McGill University Health Centre, Montreal, QC; † Department of Cardiology, McGill University Health Centre, Montreal, QC

**Keywords:** 5-Fluorouracil, capecitabine, cardiotoxicity, chemotherapy

## Abstract

Capecitabine, an oral prodrug of 5-fluorouracil (5fu), has been integrated into the management of multiple cancer types because of convenience of administration and efficacy comparable with 5fu. Cardiotoxicity induced by 5fu—in particular angina—has been well described in the literature, but reports of adverse cardiac events with capecitabine are also emerging. The mechanism underlying 5fu cardiotoxicity has long been thought to result from coronary vasospasm, but animal-model studies and patient echocardiographic findings both suggest a cardiomyopathic picture. Although 5fu cardiotoxicity is often reversible and can be managed supportively, presentations that are more severe—including arrhythmias, acute ischemic events, and cardiogenic shock—have been documented. In this report, we describe the case of a patient who ultimately required a pacemaker after developing symptomatic bradycardia and sinus arrest while receiving capecitabine for colon cancer.

## 1. CASE DESCRIPTION

A 75-year-old man with known hypertension, diabetes, and dyslipidemia was diagnosed with a stage iiib rectosigmoid adenocarcinoma in July 2007. He underwent a lower anterior resection, and 12 cycles of modified folfox-6 [oxaliplatin 85 mg/m^2^, 5-fluorouracil (5fu) 400 mg/m^2^ bolus, and leucovorin (lv) 400 mg/m^2^, followed by 5fu 2.4 g/m^2^ by continuous infusion over 46 hours] adjuvant chemotherapy were planned.

Following chemotherapy cycle 7 in late January 2008, the patient developed atrial fibrillation, and he was admitted to the coronary care unit (ccu) at the Montreal General Hospital. At that time, echocardiography revealed an ejection fraction of 55%–60%, mild left ventricular diastolic dysfunction, and mild mitral regurgitation, with otherwise normal readings. He was converted to sinus rhythm on oral sotalol (80 mg twice daily), with the plan to initiate anticoagulation upon completion of chemotherapy. One week post discharge, he developed an upper extremity deep venous thrombosis on the side of his implanted catheter, and he was started on dalteparin. Two weeks later, his chemotherapy was changed to capecitabine monotherapy at 1500 mg/m^2^ twice daily.

**FIGURE 1:**
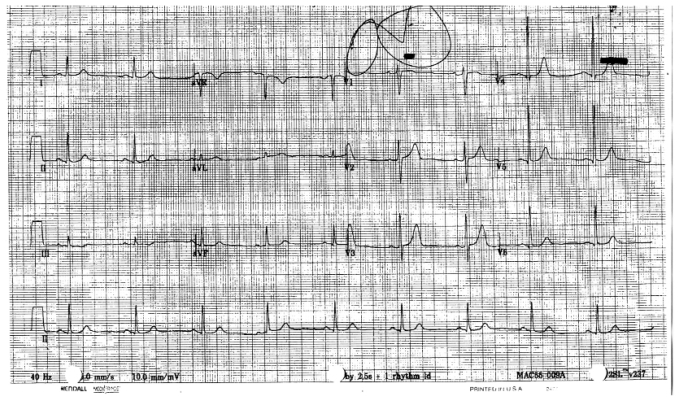
Electrocardiogram taken upon initial presentation of the patient to the emergency department.

About 6 days after starting capecitabine, the patient had a syncopal episode lasting approximately 2 minutes. He recovered spontaneously and, after some transient disorientation, returned to his baseline mental status. A few hours later, he experienced two presyncopal episodes accompanied by flushing and dizziness. He went to the emergency department, where he was found to have bradycardia at 54 bpm, although he was hemodynamically stable with a blood pressure of 140/79 mmHg. Initial blood work did not reveal any electrolyte abnormalities or tropinemia. An electrocardiogram demonstrated sinus bradycardia (Figure 1).

**FIGURE 2:**
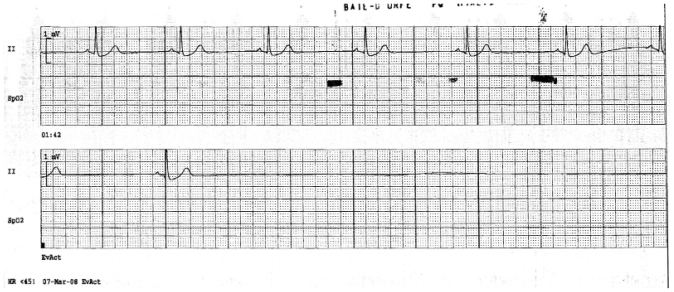
Electrocardiogram showing development of sinus bradycardia followed by sinus arrest 6 hours after the patient took capecitabine.

While on telemetry, the patient had a witnessed episode of presyncope associated with a pulse of 30–40 bpm and systolic pressure between 160– 170 mmHg. With the exception of sotalol, he received the evening doses of all of his other medications (capecitabine, metformin, venlafaxine, and valsartan) and was kept in the emergency department overnight for monitoring. Approximately 6 hours later, he had a sinus arrest of 15 s, from which he spontaneously recovered (Figure 2). Transcutaneous pacemaker pads were placed, and he was transferred to the ccu with atropine at the bedside. All medications were subsequently held.

The rest of the night was uneventful, but the following morning he had recurrent episodes of symptomatic bradycardia followed by another sinus arrest lasting 10 s. A transvenous pacemaker was inserted followed by a permanent pacemaker a few days later. Sotalol was resumed, and he was discharged without any further complications. He remains in clinical remission from his malignancy, and no further chemotherapy is planned.

## 2. DISCUSSION

Coronary vasospasm resulting in angina and even myocardial infarction is a well-known adverse effect of 5fu. Although cardiotoxicity is often reversible with discontinuation of 5fu and application of supportive care 1, the condition has the potential to be fatal. Reported overall mortality rates range from 2.2% to as high as 13.3% 2. The incidence of 5fu-induced cardiotoxicity in the literature ranges from 1.2% to 18%, although its true incidence may actually be higher, given that silent, reversible ST segment deviations on electrocardiography are known to occur 3,4. Other manifestations of 5fu-induced cardiotoxicity include heart failure, hyper- or hypotension, cardiomyopathy, arrhythmias, conduction disturbances, and cardiac arrest 5. However, these latter cardiac adverse events have been reported much less frequently.

Capecitabine is an oral prodrug that is converted to 5fu in a sequential 3-step enzymatic reaction that occurs primarily in the liver and in tumour cells. It has gained popularity because of its efficacy, ease of administration, and milder toxicity profile as compared with 5fu 6. However, as the use of capecitabine becomes more widespread, the rare but significant cardiotoxic potential of the drug is beginning to surface. In a retrospective analysis performed on studies of patients undergoing chemotherapy for metastatic breast and colon cancer, the incidence of cardiotoxicity with capecitabine was found to be comparable to that of 5fu–lv 7. Wijesinghe *et al.* 8 reported an acute coronary syndrome in a patient with no history of cardiovascular disease who had been on capecitabine for only 2 days. Kosmas *et al.* 5 documented myocardial infarction, electrocardiographic abnormalities, and ventricular extrasystoles in patients on capecitabine. Furthermore, Goldsmith *et al.* 9 recently reported exercise-induced global myocardial ischemia with an ejection fraction of 36% in a patient with normal coronary arteries and resting left ventricular function who was on capecitabine for recurrent breast cancer.

We present the first reported case of a patient who ultimately required a permanent pacemaker after developing symptomatic bradycardia followed by sinus arrest while on capecitabine. Although the patient was taking sotalol, which can itself cause bradycardia and asystole, the sotalol had been started a month before the relevant episode, during which time the patient did not experience any of the noted complications. Given that capecitabine was started less than a week before presentation, it would appear to be the most likely culprit in the absence of other medication changes at the time.

The clinical manifestations of 5fu cardiotoxicity are frequently attributed to coronary vasospasm. However, anti-vasospastic agents including nitroglycerin and calcium channel blockers have not been consistently effective at relieving symptoms of angina in affected patients 1,5,10. In a study by Freeman *et al.* 11, ergonovine challenge followed by 5fu infusion did not produce coronary vasospasm in a patient who had developed severe chest pain and ischemic electrocardiographic changes. *In vivo* studies in animal models have also provided evidence suggesting that mechanisms aside from coronary vasospasm may be responsible. Tsibiribi *et al.* 12 subjected rabbits to either bolus or infusional 5fu. Rabbits that received the bolus suffered massive hemorrhagic myocardial infarcts with evidence of proximal coronary vasospasm. In contrast, animals in the infusion group demonstrated histologic changes consistent with toxic myocarditis. In addition, echocardiographic studies in patients with 5fu cardiotoxicity have revealed decreased ejection fraction and significant global or regional left ventricular dysfunction consistent with a cardiomyopathic picture 13,14.

The metabolites of 5fu have been implicated in mediating cardiac damage, in particular fluoroacetate (fac), which is known to be directly myocardiotoxic 15. Fluoroacetate is converted into an inhibitor of citrate metabolism 16 and interestingly, intramyocardial citrate accumulation has been found in guinea pig recipients of 5fu 17. A report by de Forni *et al.* 18 also noted a trend toward increased urinary excretion of fac during 5fu administration in 14 patients, including 6 who developed cardiotoxicity, although there was no strict temporal relationship with symptom onset. Other hypothesized mechanisms that are undergoing further investigation include the possibility of an autoimmune reaction, endothelial damage, and a procoagulable effect of 5fu 19,20.

The route of administration, the dose intensity, and the schedule of 5fu also appear to influence the development of cardiotoxicity. In studies by Jensen *et al.* 2 and Kosmas *et al.* 5, a higher frequency of symptoms was recorded with continuous 5fu infusions (24 hours) than with shorter (<3 hours) infusions. Given that the pharmacokinetics of capecitabine mimic a continuous 5fu infusion, it is not surprising that the frequency of cardiac symptoms noted with capecitabine is similar to that with the 24-hour infusion 5. Higher doses of 5fu were associated with earlier symptom onset, and a weak trend toward longer symptom duration with capecitabine than with intravenous 5fu was also observed 2.

Drug pharmacokinetics and pharmacodynamics should also be considered to be additional factors that may predispose patients to developing cardiotoxicity with capecitabine. Lethal gastrointestinal toxicities have been reported with capecitabine, which have been attributed to a deficiency of dihydropyridine dehydrogenase (dpd), the first enzymatic step in 5fu catabolism 21. Cytidine deaminase (cda) is a critical enzyme for the activation of capecitabine in hepatocytes and tumour cells. Excessive levels of cda resulting in overmetabolism of capecitabine into 5fu could explain the development of severe toxicities even in patients who are not dpd-deficient 22. Interestingly, geographic differences in tolerance to capecitabine have been documented, with the highest rates of toxicity occurring in American patients and the lowest in East Asian patients 23. Although cultural and dietary differences, in addition to regional variations in the reporting of toxicities, may have contributed to these observations, polymorphisms in the genes responsible for 5fu and capecitabine metabolism may have been a factor as well 23.

The patient presented here received capecitabine after experiencing a cardiac event (atrial fibrillation) while on 5fu. It is unclear whether his atrial fibrillation was a result of exposure to 5fu. Although the patient had risk factors for coronary artery disease, he never exhibited clinical, electrocardiographic, or laboratory abnormalities to suggest that ischemia contributed to his conduction abnormalities. Nevertheless, this case raises several important issues, the first of which is how one might identify patients who may be at risk of developing cardiotoxicity on 5fu. In some studies, a history of heart disease was a risk factor for experiencing a higher incidence and grade of cardiotoxicity 2,24,25. Conversely, Tsibiribi *et al.* reported symptoms of cardiotoxicity in 16 patients with no prior cardiac history 26. Other risk factors include advanced age, renal insufficiency 2, and mediastinal irradiation (which induces coronary artery intraluminal hyperplasia and collagen deposition 27). In view of what is being learned about the pharmacokinetics of capecitabine and 5fu, the measurement of key enzymes such as dpd and cda might eventually become important in helping to predict—and hopefully spare patients from—severe toxicities resulting from these drugs.

Another issue relates to monitoring while on therapy, with the goal of detecting preclinical markers of toxicity. Holubec *et al.* 28 recently evaluated the utility of measuring brain natriuretic peptide (bnp) and troponin I (tni) in patients receiving 5fu-based chemotherapy for colorectal cancer. In that study, 57% of patients were found to have laboratory evidence of ischemia and heart failure. Upon questioning, patients recalled transient symptoms of angina, dyspnea, and edema, although the timing of biomarker elevation and symptom onset was not precisely defined. Given the high incidence of cardiotoxicity that was observed, the authors proposed that all patients for whom 5fu chemotherapy is planned should undergo baseline electrocardiographic screening or, in those with prior cardiac history, echocardiography. They also suggested that tni and bnp be monitored throughout the course of chemotherapy as surrogate markers of cardiotoxicity.

Finally, there is the question of rechallenging and giving prophylaxis to patients who have experienced cardiotoxicity but who nevertheless stand to benefit from 5fu. Cianci *et al.* 29 successfully re-administered 5fu to 3 patients who developed symptoms of angina during the initial infusion— albeit using reduced doses administered together with prophylactic trans-epidermal nitroglycerin. In another case series of 6 patients who developed transient asymptomatic bradycardia on infusional 5fu, 4 patients were able to continue treatment, but 2 had to change their regimen because of persistent and recurring bradycardia with subsequent cycles 30. Patients in series reporting more severe cardiotoxicity, such as myocardial infarction, were not rechallenged 7,31. In such patients, for whom a rechallenge of 5fu or capecitabine seems too risky, raltitrexed appears to be a viable substitute 25.

## 3. CONCLUSIONS

In summary, cardiotoxicity induced by 5fu or capecitabine has multiple manifestations and may occur more frequently than previously reported. Although this cardiotoxicity is transient and often reversible, it has the potential to cause serious morbidity and even mortality. There are currently no published guidelines for screening, monitoring, and prophylaxis. Decisions with respect to managing and rechallenging patients who have developed cardiotoxicity on 5fu must be made on an individual basis. Oncologists and cardiologists alike must maintain a heightened awareness of the possibility of this phenomenon and collaborate closely to ensure patient safety.

## 4. CONFLICT OF INTEREST

The authors declare no conflicts of interest.
